# VraSR and Virulence Trait Modulation during Daptomycin Resistance in Methicillin-Resistant *Staphylococcus aureus* Infection

**DOI:** 10.1128/mSphere.00557-18

**Published:** 2019-02-13

**Authors:** Agustina Taglialegna, Maria C. Varela, Roberto R. Rosato, Adriana E. Rosato

**Affiliations:** a Department of Pathology and Genomic Medicine, Center for Molecular and Translational Human Infectious Diseases Research, Houston Methodist Research Institute, Houston, Texas, USA; b Houston Methodist Cancer Center, Houston Methodist Hospital, Houston, Texas, USA; University of Nebraska Medical Center

**Keywords:** daptomycin, MRSA, VraSR, virulence

## Abstract

Methicillin-resistant Staphylococcus aureus (MRSA) threatens human health in hospital and community settings. The lipopeptide antibiotic daptomycin (DAP) is a frequently used treatment option for MRSA infection. DAP exposure can cause bacterial resistance because mutations are induced in genes implicated in cell membrane and cell wall metabolism. Adaptations aimed at surviving antimicrobial pressure can affect bacterial physiology and modify *in vivo* aptitude and pathogenesis. In this study, clinical DAP-susceptible (DAP^s^) and DAP-resistant (DAP^r^) MRSA isolates were used to investigate associations between DAP resistance and staphylococcal virulence. We previously found that VraSR is a critical sensor of cell membrane/wall homeostasis associated with DAP acquisition during MRSA infection. The present study found that DAP^r^ CB1634 and CB5014 MRSA strains with *vraSR* upregulation were less virulent than their susceptible counterparts, CB1631 and CB5013. Differential gene-transcription profile analysis revealed that DAP^r^ CB1634 had decreased *agr* two-component system expression, virulence factors, and highly suppressed hemolysis activity. Functional genetic analysis performed in DAP^r^ CB1634 strains using *vraSR* inactivation followed by gene complementation found that *vraSR* acted as a transcriptional *agrA* regulator. These results indicated that VraSR has a broad range of regulatory functions. VraSR also appeared to affect DAP^r^ adherence to epithelial cells, which would affect DAP^r^ strain colonization and survival in the host. The correlation between DAP resistance and decreased virulence was also found in the CB5013 (DAP^s^) and CB5014 (DAP^r^) pair. Taken together, these findings are the first evidence that DAP resistance and MRSA virulence are tightly connected and involve compromised expression of regulatory and virulence determinants.

**IMPORTANCE** Methicillin-resistant S. aureus continues to develop resistance to antimicrobials, including those in current clinical use as daptomycin (DAP). Resistance to DAP arises by mutations in cell membrane and cell wall genes and/or upregulation of the two-component VraSR system. However, less is known about the connection between the pathogen and virulence traits during DAP resistance development. We provide new insights into VraSR and its regulatory role for virulence factors during DAP resistance, highlighting coordinated interactions that favor the higher persistence of MRSA DAP-resistant strains in the infected host.

## Abstract (precis)

Methicillin-resistant S. aureus continues to develop resistance to antimicrobials, including those in current clinical use as daptomycin (DAP). Resistance to DAP arises by mutations in cell membrane and cell wall genes and/or upregulation of the two-component VraSR system. However, less is known about the connection between the pathogen and virulence traits during DAP resistance development. We provide new insights into VraSR and its regulatory role for virulence factors during DAP resistance, highlighting coordinated interactions that favor the higher persistence of MRSA DAP-resistant strains in the infected host.

## INTRODUCTION

Staphylococcus aureus is a significant and ubiquitous opportunistic pathogen. The multidrug-resistant pathogen methicillin-resistant S. aureus (MRSA) is a major concern for public health in hospital and community settings and is associated with the development of numerous diseases (1). These diseases range from skin and soft tissue infections to severe life-threatening infections (e.g., pneumonia, endocarditis, and bacteremia) (2). Prevention of MRSA infection has improved, but infections caused by this pathogen remain challenging. The anti-MRSA antibiotics approved for different infections (e.g., complicated skin structure infections, bacteremia, and pneumonia) include vancomycin, linezolid, telavancin, ceftaroline, and daptomycin (DAP) (3).

DAP is a cyclic anionic lipopeptide that shares structural similarities with cationic antimicrobial peptides (CAMPs), a group of molecules produced by mammalian innate immune systems (4). DAP molecules first form micelles in the presence of physiological calcium concentrations. Next, phospholipid phosphatidylglycerol (PG) induces a structural transition in the DAP-calcium complex, allowing its binding to the cytoplasmic membrane (5), causing membrane depolarization, homeostasis imbalance, and cell death (4).

DAP-resistant S. aureus clinical isolates have been isolated from patients treated with DAP and other antibiotics (e.g., vancomycin) (6, 7). Although DAP resistance is rare, treatment failure occurs in more than 20% of the cases of resistance (8, 9) and still represents a challenge when encountered (10–12).

To resist DAP activity, the bacteria must impede the drug from reaching the cell membrane or penetrating it (5). The main factors described involving resistance to DAP, among other possible processes, include (i) production of a more positively charged cell surface to prevent DAP-Ca^2+^ insertion through electrostatic repulsion (13, 14), (ii) alteration of membrane fluidity by changing phospholipid content and asymmetry (13, 15, 16), (iii) decreased autolysis and increased thickening of the cell wall (17–19), and (iv) physiological and metabolic adaptations directed to increase the carbon flow to the synthesis of precursors needed for cell wall biosynthesis (18). Underlying these mechanisms are different nonsynonymous mutations in genes involved in the regulation of cell membrane structure and function, notably *mprF*, which is the most frequently described mutation in clinical DAP-resistant strains (14, 20–22). Other mechanistically relevant mutations can include those in cell wall-associated components (23, 24).

The success of a pathogen in overcoming a given antimicrobial therapy and continuing to spread during infections depends not only on the intrinsic and acquired resistance to the drug but also on additional factors, such as the resistance fitness costs, the pathogenicity of the strain, and the host conditions. The interplay between these mechanisms is poorly understood. Many studies have described a relationship between resistance mechanisms and virulence in several Gram-negative bacterial species, such as Pseudomonas aeruginosa (25–27), Acinetobacter baumannii (28–30), Escherichia coli (31), and Klebsiella pneumoniae (32, 33). For multidrug-resistant S. aureus, there is evidence demonstrating a tight connection between resistance to β-lactams, vancomycin, and glycopeptides and the pathogenicity of the MRSA strains (34–37). However, the impact of acquiring DAP resistance in clinical S. aureus and its correlation with pathogenicity and virulence have not been deeply explored.

We previously found that *mprF* mutation is not the only factor that determines DAP resistance. We provided functional evidence that upregulation of *vraSR* is a key factor associated with DAP and that inactivation results in increased DAP susceptibility. We also found that VraSR is a critical regulator of cell membrane homeostasis in response to alteration of membrane surface charges and reorganization of cell division proteins associated with cell wall synthesis (38). The accessory gene regulator (*agr*) is an important virulence regulator during S. aureus infection. RNA III is the effector of the system known to upregulate the expression of toxins and to downregulate genes encoding cell surface-associated proteins (39). The *agr* operon mutation has been commonly reported for VISA (vancomycin-intermediate S. aureus); *agr* dysfunction in the absence of mutation has been also described. The loss of *agr* that occurs frequently in clinical isolates enhances the survival of S. aureus during DAP treatment. This result compares with the rapid killing of wild-type S. aureus strains (40). In the present study, we used *in vitro* and *in vivo* experiments and found that acquisition of DAP resistance and virulence in MRSA is a tightly connected and regulated mechanism that includes a cross-talk regulatory pathway between *vraSR* and *agr*. This process may contribute to the persistence of DAP-resistant strains during infection.

## RESULTS

### Acquisition of DAP resistance impacts MRSA strain pathogenicity.

**FIG 1:**
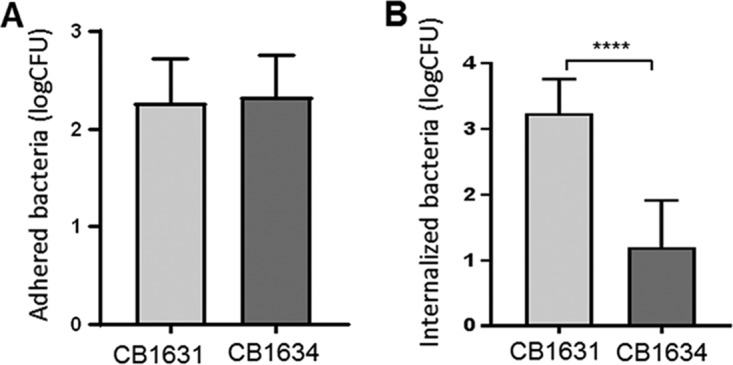
S. aureus susceptibility to DAP correlates with the virulence of MRSA strains. (A) Adhesion of S. aureus DAP^s^ CB1631 and DAP^r^ CB1634 to A459 human epithelial cells. (B) Internalization of S. aureus DAP^s^ CB1631 and DAP^r^ CB1634 into human A459 epithelial cells. Data represent the mean and standard deviation from three independent experiments. Statistically significant differences were determined using an unpaired Student *t* test (****, *P* < 0.0001).

In previous studies, we explored the detailed mechanistic basis of DAP resistance in a set of clinical isogenic DAP^s^/DAP^r^ strains (11, 38). However, the effect of acquiring DAP resistance on the virulence of the strain has not been examined. We used two of the previously characterized isogenic MRSA clinical strains, DAP^s^ CB1631 and DAP^r^ CB1634, which were isolated from a patient who had a DAP therapy failure (11) (Table 1). We evaluated the capacity of both strains to adhere to and invade the human epithelial cell line A529. While no significant differences in adhesion were observed between the two strains (Fig. 1A), when the strains where assessed for their ability to internalize into the A259 cells (Fig. 1B), DAP^s^ CB1631 was more effective than its resistant CB1634 counterpart. This finding is in accordance with increased expression of *fnbAB* and *isdABDE* observed in CB1631 (Table 2), suggesting that the DAP^s^ strain elicits more pronounced invasion traits than DAP^r^ CB1634.

**TABLE 1:** Bacterial strains used in this study and their MIC values obtained using the Etest^*a*^

Strain or plasmid	Description	DAP MIC (µg/ml)	Reference or source
Strains			
S. aureus			
N315	Hospital-acquired methicillin-resistant SCC*mec* type II	0.125	60
ATCC 29213	MSSA, standard strain for CLSI antimicrobial susceptibility testing	0.125	61
Newman	MSSA, isolated from a human infection	0.25	62
KVR	N315 Δ*vraSR*::*cat*		60
CB1631	DAP^s^, SCC*mec* type II	0.25	41
CB1634	DAP^r^ isogenic to CB1631	4	41
CB1634+*agr*	CB1634 + expressing *psk265* full-length *agr*	0.094	This study
CB5013	DAP^s^, SCC*mec* type II	0.25	41
CB5014	DAP^r^, isogenic to CB5013	4	41
CB1634Δ*vraSR*	CB1634 Δ*vraSR*::*cat*	0.25	This study
CB1634Δ*vraSR*+Δ*vraSR*	CB1634 Δ*vraSR*::*cat* pVRASR-2	2	This study
CB5014Δ*vraSR*	CB5014 Δ*vraSR*::*cat*	0.25	This study
CB5014Δ*vraSR*+Δ*vraSR*	CB5014 Δ*vraSR*::*cat* pVRASR-2	2	This study
S. epidermidis Y1		0.25	This study
Plasmids			
pCR-XL-2 TOPO	Cloning vector, Amp^r^ Kan^r^		ThermoFisher
S. aureus RN4220(pVRASR-2)	Entire *vraS*/*vraR* cloned into pAW8-*tet*		39

a Abbreviations: *cat*, chloramphenicol resistant; Tet^r^, tetracycline resistant; Amp^r^, ampicillin resistant; Kan^r^, kanamycin resistant.

**TABLE 2:** Gene expression analysis of S. aureus DAP^s^ CB1631 compared with DAP^r^ CB1634 using RNA-seq

ORF	Gene	Product or function	Fold change
SA1844	*agrA*	Accessory gene regulator A	4.501
SA1842	*agrB*	Accessory gene regulator B	1.984
SA1843	*agrC*	Accessory gene regulator C	2.218
SAS066	*agrD*	Accessory gene regulator R	2.424
SA2457	*capA*	Capsular polysaccharide biosynthesis protein Cap5A	1.359
SA0145	*capB*	Capsular polysaccharide biosynthesis protein Cap5B	4.856
SA0146	*capC*	Capsular polysaccharide biosynthesis protein Cap5C	1.444
SA0147	*capD*	Capsular polysaccharide biosynthesis protein Cap5D	2.363
SA0148	*capE*	Capsular polysaccharide biosynthesis protein Cap5E	2.672
SA0149	*capF*	Capsular polysaccharide biosynthesis protein Cap5F	2.425
SA0150	*capG*	Capsular polysaccharide biosynthesis protein Cap5G	1.798
SA0151	*capH*	Capsular polysaccharide biosynthesis protein Cap5H	3.642
SA0152	*capI*	Capsular polysaccharide biosynthesis protein Cap5I	1.641
SA0153	*capJ*	Capsular polysaccharide biosynthesis protein Cap5J	1.288
SA0154	*capK*	Capsular polysaccharide biosynthesis protein Cap5K	1.99
SA0155	*capL*	Capsular polysaccharide biosynthesis protein Cap5L	1.213
SA0156	*capM*	Capsular polysaccharide biosynthesis protein Cap5M	1.217
SA0157	*capN*	Capsular polysaccharide biosynthesis protein Cap5N	2.879
SA0159	*capP*	Capsular polysaccharide biosynthesis protein Cap5P	1.259
SA0742	*clfA*	Clumping factor A, fibrinogen binding protein	−1.977
SA2423	*clfB*	Clumping factor B, fibrinogen binding protein	1.039
SA0222	*coa*	Staphylocoagulase precursor	2.458
SA2291	*fnbA*	Fibronectin binding protein A	1.742
SA2290	*fnbB*	Fibronectin binding protein B	2.692
SA0309	*geh*	Lipase	1.274
SA1756	*hlb*	Truncated β-hemolysin	3.075
SAS065	*hld*	δ-Hemolysin	8.235
SA2207	*hlgA*	γ-Hemolysin component A	1.412
SA2209	*hlgB*	γ-Hemolysin component B	2.195
SA2208	*hlgC*	γ-Hemolysin component C	1.476
SA2356	*isaA*	Immunodominant antigen A	2.629
SA0977	*isdA*	Iron-regulated surface determinant protein A	2.582
SA0976	*isdB*	Iron-regulated surface determinant protein B	2.841
SA0979	*isdD*	Iron-regulated surface determinant protein D	3.817
SA0980	*isdE*	Iron-regulated surface determinant protein E	2.702
SA1637	*lukD*	Leukotoxin	3.537
SA1638	*lukE*	Leukotoxin	3.515
SA0661	*saeR*	Response regulator SaeR	4.824
SA0660	*saeS*	Sensor histidine kinase SaeS	3.078
SA2206	*sbi*	Immunoglobulin G binding protein	4.546
SA0519	*sdrC*	Serine-aspartate repeat-containing protein C, fibrinogen binding protein	1.171
SA0520	*sdrD*	Serine-aspartate repeat-containing protein D, fibrinogen binding protein	1.316
SA0521	*sdrE*	Serine-aspartate repeat-containing protein E, fibrinogen binding protein	1.165
SA1869	*sigB*	RNA polymerase sigma factor	1.066
SA0107	*spa*	Protein A	−1.264
SA1631	*splA*	Serine protease	4.269
SA1630	*splB*	Serine protease	2.244
SA1629	*splC*	Serine protease	1.147
SA1628	*splD*	Serine protease	3.059
SA2093	*ssaA*	Staphylococcal secretory antigen	2.6
SA1700	*vraR*	Response regulator VraR	−3.021
SA1701	*vraS*	Sensor protein VraS	−1.816
SA0018	*walK*	Sensor protein kinase WalK (VicK, YycG)	−1.122
SA0017	*walR*	Response regulator WalR (VicR, YycF)	−1.289

### DAP^r^ MRSA strains exhibit decreased transcription of virulence genes.

**FIG 2:**
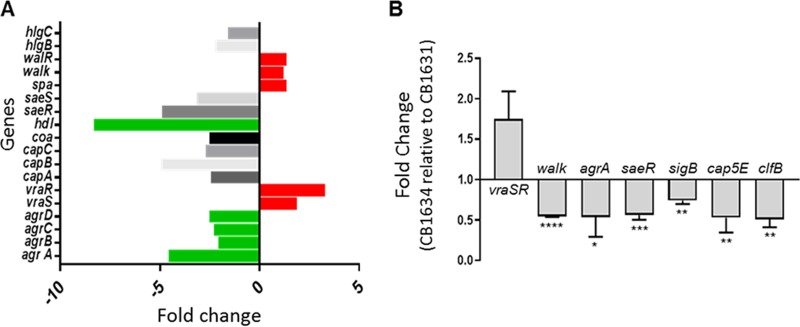
Gene expression analysis. (A) RNA-seq expression analysis comparing CB1634 with CB1631, expressed in fold changes. (B) Quantification of the mRNA expression of regulatory and virulence genes in the S. aureus CB1634 strain relative to its parental CB1631 strain using qRT-PCR. Statistically significant differences were determined using an unpaired Student *t* test (*, *P* < 0.05; **, *P* < 0.01; ***, *P* < 0.001; ****, *P* < 0.0001). Genes upregulated are denoted in red, and genes downregulated are shown in green.

To interrogate whether differences in gene expression could reflect the potential factors linking virulence and DAP resistance, we analyzed the transcription levels of several major staphylococcal virulence factors in DAP^s^ CB1631 and DAP^r^ CB1634 strains using RNA-seq. Master gene regulators (*agrA*, *saeR*, and *saeS*), which control the global expression of multiple virulence factors, were upregulated in the CB1631 strain (Fig. 2A and Table 2). The genes with the highest upregulation were those coding for cytolytic proteins (*hld*, *hlb*, *lukD*, and *lukE*), serine proteases (*splA*, *splC*, and *splD*), coagulases (*coa*), surface adhesins important for host colonization (*isdA*, *isdB*, *isdD*, and *isdE*), capsular biosynthesis proteins (*cap5*), and innate immune response evasion factors (*sbi*). In contrast, the two-component system kinase sensor *vraS* and its response regulator *vraR* were downregulated, consistent with the observed DAP^s^ phenotype of CB1631 (41). Notably, two important adhesins (*spa* and *clfA*) were downregulated in DAP^s^ CB1631. To validate the RNA-seq results, we performed reverse transcription-quantitative PCR (qRT-PCR) for most of the virulence genes and master regulators. As shown in Fig. 2B, *agrA*, *saeR*, and *sigB* regulators showed significantly decreased levels of transcription in DAP^r^ CB1634 compared with the DAP^s^ strain while transcription levels of *vraSR* mRNA were increased.

Together, these results suggest that the acquisition of DAP resistance impacts the transcriptional profile and regulatory pathways of MRSA, which could influence the pathobiology of S. aureus.

### Decreased hemolysis production and virulence in DAP^r^ CB1634.

**FIG 3:**
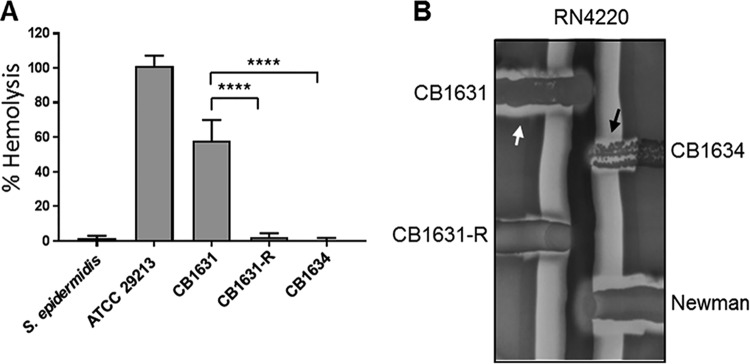
Hemolysis of RBCs. (A) Rabbit erythrocytes were incubated with an equal volume of bacterial supernatants, and hemolysis was measured spectrophotometrically using absorbance at 540 nm. A standard curve was used to determine the percent hemolysis. S. epidermidis was used as a nonhemolytic control; S. aureus ATCC 29213 was used as a positive control. Data represent the means and standard deviations from at least three independent experiments. Statistically significant differences were determined using a one-way ANOVA; a Bonferroni *a posteriori* test was performed (****, *P* < 0.0001). (B) δ-hemolysin activity was present in CB1631, CB1634, and, to a lesser extent, in CB1631-R (black arrow). α-hemolysis was present in CB1631 (white arrow).

A feature of highly virulent strains is their ability to lyse red blood cells (RBCs) by secreting hemolysins. As mentioned previously, we found decreased transcription levels of *agrA* and *saeR* and a mutation in *saeR* in the DAP^r^ S. aureus CB1634 strain. These two regulators are some of the primary activators of staphylococcal hemolysins. To further confirm the invasiveness of DAP^s^ strains, we performed a hemolytic assay using rabbit RBCs (Fig. 3A). Staphylococcusepidermidis and S. aureus ATCC 29213 were used as nonhemolytic and hemolytic controls, respectively. In this experiment, we included the *in vitro*-generated CB1631-R strain. As shown in Fig. 3A, the S. aureus DAP^s^ CB1631 strain elicited significantly more hemolysis than its *in vivo* CB1634 and *in vitro*-generated CB1631-R DAP^r^ counterparts. These observations were followed by the measuring of hemolysis production in both DAP^r^ and DAP^s^ strains. As illustrated in Fig. 3B, the DAP^s^ CB1631 strain produced α-hemolysin and δ-hemolysin, which is demonstrated by the clearing zone at the intersection of the β-hemolysis halo of the S. aureus RN4220 streak; similar effects were observed with the positive-control S. aureus Newman strain. For DAP^r^ CB1634, there was only δ-hemolysis and absence of α-hemolysin. Similarly, in the *in vitro*-obtained DAP^r^ mutant CB1631-R, its hemolysin production was markedly diminished compared with the parental strain (Fig. 3B). Thus, these results suggested a potential connection between levels of DAP resistance acquired either *in vitro* or *in vivo* and decreased pathogenicity in the host.

### VraSR regulatory cross talk with *agr* determines increases in virulence and hemolysis.

Cameron et al. recently described an association between VraSR and VISA strain virulence (42). They demonstrated the capacity of VraSR to modulate S. aureus virulence by binding the P2-P3 intergenic region of the *agr* promoter, indicating that when S. aureus is subject to vancomycin induction, VraR binds and inhibits the function of the Agr quorum sensing system, causing reductions in the virulence of VISA/hVISA strains (42, 43). Given our previous findings revealing (i) that VraSR is a key factor during DAP resistance and (ii) the defective expression of *agr* found in CB1634, we postulated that VraSR may transcriptionally regulate *agr* expression in CB1634.

**FIG 4:**
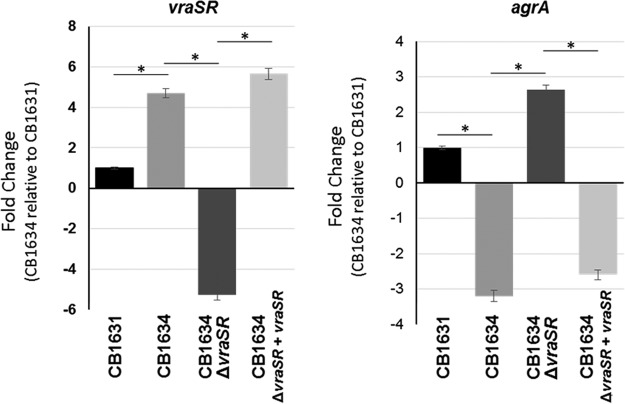
Quantitation of *vraSR* and *agrA* mRNA using real-time RT-PCR. RNA was prepared from cells of DAP^s^ CB1631, DAP^r^ CB1634, CB1634*ΔvraSR*, and complemented mutant CB1634*ΔvraSR+vraSR* strains collected during the exponential phase of growth. Relative fold change values of specific *vraSR* mRNA are shown on the vertical axis; 16S rRNA was used as an internal control (*, *P* < 0.001).

To test this hypothesis, we evaluated mRNA expression levels of both *agrA* and *vraSR* using real-time reverse transcription-PCR (RT-PCR) analysis (Fig. 4), showing consistency with results of RNA-seq analysis (Fig. 2A), i.e., decreased expression of *agrA* in CB1634 when *vraSR* was upregulated (Fig. 4). These effects were further analyzed using a *vraSR* mutant generated in the CB1634 strain, as described in Materials and Methods. We found that inactivation of *vraSR* (CB1634Δ*vraSR*) resulted in increased *agr* expression, while *vraSR* complementation (CB1634Δ*vraSR*+*vraSR*) reversed these effects and reduced *agrA* to levels comparable to those seen in the CB1634 strain. Similar results were obtained when additional DAP^s/r^ strains (e.g., CB5013/CB5014) and corresponding mutant/transcomplemented strains were examined (data not shown).

**FIG 5:**
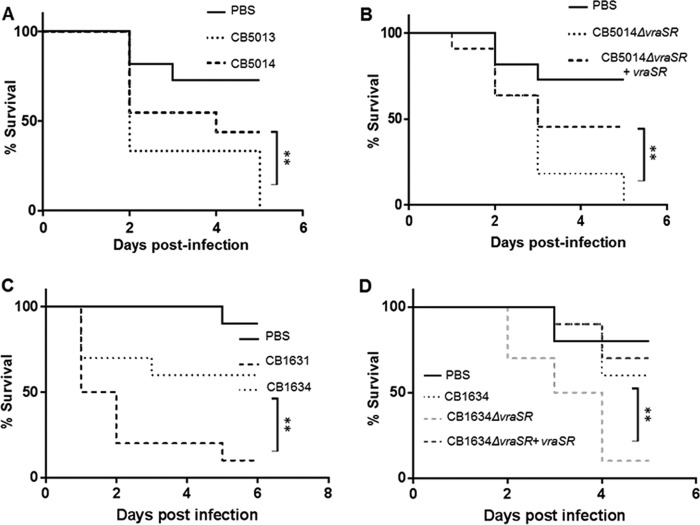
G. mellonella infection with DAP^s/r^ and derivative strains. Groups of larvae (10/group) were inoculated with 10 µl PBS (uninfected control group) or bacterial suspension containing 1.5 × 10^6^ CFU/ml DAP^s^ CB5013 and DAP^r^ CB5014 (A) and its corresponding mutant and complemented strains, CB5014*ΔvraSR* and CB5014*ΔvraSR*+*vraSR* (B), into the last proleg and incubated at 37°C. Worms were checked daily, and any deaths were recorded, for a total of 10 days. A minimum of three independent experimental replicates were performed for each experiment. Similar analyses were performed with DAP^s^ CB1631 and DAP^r^ CB1634 (C) and its corresponding mutant and complemented strains, CB1634*ΔvraSR* and CB1634*ΔvraSR*+*vraSR* (D). Survival data were plotted using the Kaplan-Meier method and expressed as percentage of survival versus time. Statistically significant differences were determined using the log rank test (**, *P* < 0.01).

To evaluate the effects of *vraSR/agrA* on virulence traits, we tested the role of the aforementioned regulatory system in an *in vivo* model of Galleria mellonella infection. Groups of larvae were inoculated with a bacterial suspension containing the corresponding CB5013, CB5014, CB5014Δ*vraSR*, and CB5014Δ*vraSR*+*vraSR* strains (10^6^ bacteria/worm), as previously described (11). An uninfected control group received a PBS treatment to control for multiple injections. Worms were monitored daily, and any deaths that occurred over the next 5 days were recorded. Worms injected with PBS showed 80% to 100% survival at day 5 (Fig. 5A and B). Groups injected with the parent CB5013 strain had low survival rates (0% to 30%, day 5; Fig. 5A), while in contrast, those worms infected with the CB5014 strain had survival rates of 40% to 70% at day 5. The CB5014*ΔvraSR* strain showed a similar trend as the one observed with the CB5013 parent strain (i.e., survival rate of 0% at day 5; Fig. 5B). Following CB5014*ΔvraSR* transcomplementation (CB5014*ΔvraSR*+v*raSR*), the survival rate was higher (50% to 90%; Fig. 5B), as observed with the worms injected with the CB5014 strains (Fig. 5A). Similar results were obtained with worms infected with CB1634, CB1634, CB1634Δ*vraSR*, and CB1634Δ*vraSR*+*vraSR* strains (10^6^ bacteria/worm; Fig. 5C and D).

**FIG 6:**
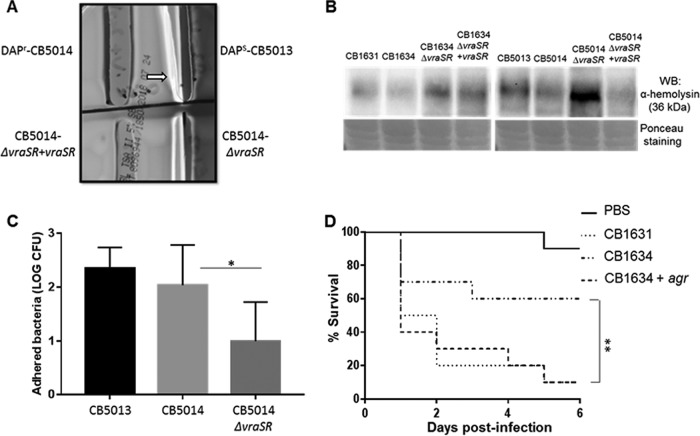
Analysis of virulence factors of DAP^r^ CB5014 and DAP^r^ CB1634 and derivative strains. (A) Hemolysis in blood agar plates of CB5013, CB5014, mutant CB5014*ΔvraSR*, and transcomplemented CB5014*ΔvraSR*+*vraSR*. (B) Western blot analyses of α-hemolysin in supernatants collected and concentrated from CB1631, CB1634, CB1634*ΔvraSR*, CB1634*ΔvraSR+vraSR*, CB5013, CB5014, CB5014*ΔvraSR*, and CB5014*ΔvraSR*+*vraSR* derivative strains. Ponceau staining was used as a loading control. α-hemolysis was higher in CB1631, CB5013, CB1634Δ*vraSR*, and CB5014Δ*vraSR*; low levels of alpha-toxin are seen in CB1634, CB5014, and complemented strains CB1634*ΔvraSR*+*vraSR* and CB5014*ΔvraSR*+*vraSR*. (C) Adhesion of S. aureus DAP^s^ CB5013 and DAP^r^ CB5014 and its corresponding mutant CB5014Δ*vraSR* to A459 human epithelial lung cells. Reduced adhesion was found in CB5014Δ*vraSR* compared with its parent DAP^r^ CB5014 counterpart (*, *P* < 0.01). (D) *G. mellonella* infection of groups of larvae (10/group) inoculated with PBS and bacterial suspension containing 1.5 × 10^6^ CFU/ml DAP^s^ CB1631, DAP^r^ CB1634, and its overexpressed *agr* derivative CB1634+*agr.* Survival data were plotted using the Kaplan-Meier method and expressed as percentage of survival versus time. Statistically significant differences were determined using the log rank test (**, *P* < 0.01).

Taking into account the observations showing differences between DAP^s/r^ strains in terms of hemolysis (Fig. 2), we further investigated whether *vraSR* may have affected α-hemolysin production. To test this hypothesis, Western blot analysis using a specific anti-α-hemolysin antibody was performed with lysates from both DAP^r^ CB1634 and CB5014 strains and the latter’s corresponding *vraSR* mutant and transcomplemented strains, CB5014Δ*vraSR* and CB5014Δ*vraSR+vraSR.* As depicted in Fig. 6B, the DAP^s^ CB1631 strain produced α-hemolysin to a greater extent than its DAP^r^ counterpart CB1634 (Fig. 6B, lanes 1 and 2). Inactivation of *vraSR* in DAP^r^ CB1634 (CB1634*ΔvraSR*) did correlate with increased α-hemolysin levels, similar to the DAP^s^ CB1631 strain (lane 3), while *vraSR* transcomplementation (CB1634*ΔvraSR*+*vraSR*) showed similar levels as those seen in DAP^r^ CB1634, i.e., decreased levels compared to CB1634*ΔvraSR*. Similar observations were made between strains CB5013, CB5014, and the corresponding *vraSR* mutant and transcomplemented strains (i.e., CB5014*ΔvraSR* and CB5014*ΔvraSR+vraSR*; lanes 5 to 8, Fig. 6B).

We then analyzed whether inactivation of VraSR affected the capacity of DAP^r^ cells to adhere to A549 human lung epithelial cells, showing there was a statistically significant difference in adhesion levels between CB5014Δ*vraSR* and CB5014 (*P* < 0.01; Fig. 6C). CB5014Δ*vraSR* had low levels of adhesion to epithelial cells compared with its parent strain, CB5014. These results suggested that VraSR is associated with α-hemolysin production and that VraSR promotes the adhesion of the DAP^r^ strain to epithelial cells.

To investigate the cross talk between *vraSR* and *agr* in relation to virulence during DAP resistance, extratemporal *agr* overexpression was performed in CB1634 using the pSK265 vector containing the wild-type copy of the *agr* operon. The results shown in Fig. 6D indicated that *agr* enforced expression in CB1634 (CB1634+*agr*) determined an increase in virulence with survival percentages similar to the parental CB1631 strain. However, α-hemolysis was not restored after *agr* complementation, suggesting that that although *agr* is associated with CB1634 defects in virulence, it was not able to restore α-hemolysis production, as there was no direct effect of *agr* on hemolysis during DAP resistance (data not shown). Moreover, we ruled out, by having performed *saeR trans*complementation of *saeR* mutation in CB1634 (CB1634+p*saeRS*_WT_), that the δ-hemolysis phenotype was identical to its parental CB1634 strain and did not show restored capacity to produce α-hemolysis (data not shown). Taken together, these results suggested that virulence in DAP^r^ strains is dependent on VraSR regulatory control of *agrA*.

**FIG 7:**
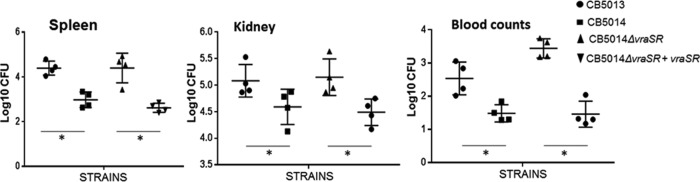
*In vivo* sepsis mouse model showing the effect of DAP susceptibility on the colonization of kidney and spleen. Groups of six mice were used. Each group (*n* = 6) was inoculated via tail injection with ∼1 × 10 to 2 × 10^7^ CFU of either the DAP^s^ CB5013 or DAP^r^ CB5014 MRSA strain grown in TSB at 37°C, 150 rpm. Mice were euthanized at 72 h postinfection. Kidneys, spleen, and whole blood were collected aseptically, homogenized (kidneys and spleen), serially diluted in PBS, and plated onto TSA plates to determine the number of viable staphylococci. Results are expressed as the logarithm of CFU per gram of organ (log CFU/g). Statistically significant differences were determined using the unpaired Student *t* test (*, *P* < 0.05).

Finally, to determine the differences in response to infection between the DAP^s^ and DAP^r^ strains in mammalian tissues, we used an established murine septicemia model. Groups of 5 to 6 mice each were inoculated via tail injection with ∼1 × 10 to 2 × 10^7^ CFU of DAP^s^ CB5013, DAP^r^ CB5014, and the corresponding *vraSR* mutant CB5014Δ*vraSR* and transcomplemented CB5014Δ*vraSR*+*vraSR* MRSA strains. After 72 h, mice were euthanized, and spleen, kidneys, and whole blood were collected aseptically, homogenized (spleen and kidneys), serially diluted in PBS, and plated onto tryptic soy agar (TSA) plates to determine the number of viable staphylococci. As depicted in Fig. 7, DAP^s^ CB5013 cells proliferated in all cases to higher values during infection compared with the DAP^r^ CB5014 isogenic strain; importantly, CB5014Δ*vraSR* displayed similar infection levels as those corresponding to CB5013, values that were significantly reduced when levels of *vraSR* were restored, i.e., CB5014Δ*vraSR*+*vraSR.* Similar results were obtained for CB1631, CB1634, and their corresponding mutant/complemented *vraSR* strains (data not shown). These results, together with the observations suggesting that MRSA DAP^s^ strains (e.g., CB5013 and CB1631) are more prone to invading mammalian cells than their DAP^r^ counterpart strains (e.g., CB1634; Fig. 1B), highlight first the attenuated *in vivo* virulence of the DAP^r^ strain and second the mechanistic role played by *vraSR* in this process.

## DISCUSSION

When facing challenging environmental conditions, bacteria can adopt diverse adaptation strategies to survive. Particularly within a host, they can adjust expression of virulence factors at any time during infection (44). In addition, if they are exposed to antimicrobial agents, they are able to generate metabolic and/or genetic changes that promote survival when exposed to a certain drug while sustaining the infection within the host (18). The correlation between resistance to several antibiotics and virulence has been demonstrated previously in different bacterial species (34, 36, 45, 46). In the present study, we analyzed how DAP resistance impacted the pathogenicity of clinically derived MRSA strains obtained from cases of DAP treatment failure. Our results showed that the *in vivo* virulence of DAP^r^ strains was notably attenuated compared with their DAP^s^ counterparts, as shown in the *in vivo* G. mellonella invertebrate model and the murine septicemia model. This finding was consistent with the observation that the counterpart DAP^s^ CB1631 strain was more hemolytic and had higher expression of different virulence determinants. This evidence was further supported by the results using an *in vitro* DAP-resistant mutant CB1631-R. A considerable rise in DAP MIC) (8 µg/ml) was associated with markedly attenuated virulence, lowered expression of virulence factors, and lowered hemolysis. These results were consistent with the results reported by Cameron et al. (45), in which clinical and *in vitro* DAP^r^ strains were less virulent but more persistent *in vivo* than their DAP^s^ progenitor strains. Similarly, DAP^r^ Streptococcus mitis strains have been shown to display reduced *in vitro* and *in vivo* virulence in an endovascular infection model; the parental DAP- susceptible strain outcompeted the DAP^r^ variant in all target organs (46). One explanation for the lowered virulence of DAP^r^ staphylococcal strains could be that bacteria are more likely to prefer to sustain a chronic persistent infection instead of invasive acute infections once they acquire resistance. In the long term, this strategy might be more beneficial for DAP^r^ strains from a fitness and survival perspective. The fitness costs of DAP resistance determine the aptitude of the strain *in vivo* and therefore influence the course and type of infection. In fact, the acquisition and maintenance of resistance is a costly process, and in order to survive the presence of antibiotics, bacteria sacrifice numerous proteins, thus losing certain abilities. In support of this hypothesis, it was shown that DAP-nonsusceptible strains isolated after DAP treatment failure had significant alterations in metabolic pathways needed to support resistance (18). In another report, *in vitro* DAP^r^ mutants of strains isolated from hospitalized patients with bloodstream infections showed decreased fitness and pathogenicity (47). Regarding our strains, Roch et al. demonstrated that DAP^s^ CB1631 and CB5013 are more fitness competent (48) and, as addressed in this work, more virulent than their isogenic DAP^r^ strains. In the present study, we found that the pattern of reduced virulence seen in clinical DAP-resistant strains also occurred in the *in vitro*-generated DAP-resistant mutant. For the CB1631-R *in vitro* mutant, the fitness repercussion of DAP resistance was even more pronounced; the strain reached a high MIC value (8 µg/ml) by incorporating nonsynonymous mutations in three different genes (*walK*, *rpoC*, and *mrpF*) in its genome. These mutations compromised growth, the expression of numerous virulence factors, and the *in vivo* pathogenic competence (data not shown).

Another important new finding of our study is that VraSR appears to have an accessory role other than sensing membrane damage in DAP-resistant strains. To understand the molecular mechanism contributing to attenuated virulence in DAP- resistant strains, we centered our attention on *vraSR* and *agr* regulators. Expression of these regulators appeared to be uniformly altered in most of our DAP^r^ strains, which ruled out an effect associated with strain background. Consistent with our findings, Pader et al. found that loss of *agr* quorum sensing, which occurs frequently in S. aureus isolates, enhances S. aureus survival during DAP treatment (40). They also demonstrated that as a mechanism of protection, defective *agr* mutants survive antibiotic exposure by releasing membrane phospholipids that bind and inactivate DAP (40).

In addition to maintaining the highly demanding process of DAP resistance, *agr* overexpression in the DAP^r^ strain (CB1634+*agr*) not only increased its virulence but also negatively affected the *in vivo* persistence (Fig. 6D). These results suggested that through downregulation of *agr*, VraSR provides DAP^r^ strains with advantageous survival traits. We found that the reduced virulence of DAP^r^ strains was reversed in their counterpart *vraSR* mutants, highlighting the role of VraSR in virulence modulation. The findings of Chang et al.in Streptococcussuis support this hypothesis (49). They described that *vraSR* has an essential role in facilitating the resistance of S. suis to killing in human blood (49). Taken together, these findings and ours indicate that VraSR may initiate a regulatory response to counteract neutrophil defense by increasing the probability of DAP^r^ strain survival in the host.

Overall, these results provide evidence that virulence and DAP resistance in MRSA are intrinsically related. It is likely that these two processes must be carefully regulated to be mutually associated. An understanding of how these mechanisms interconnect will contribute to the elucidation of the evolution of DAP^r^ strains and potentially identify methods for the prevention and treatment of life-threatening MRSA infections.

## MATERIALS AND METHODS

### Bacterial strains, culture conditions, antibiotics, and plasmids.

All strains used in this study are listed in Table 1; some of these strains were reported previously (41). Strains were grown in tryptic soy broth (TSB) (BD, Sparks, MD), Mueller-Hinton broth (MH) (BD, Sparks, MD), tryptic soy agar (TSA) (BD, Sparks, MD), TSA with 5% sheep blood (BBL, Sparks, MD), and MH agar (MHA) (BD, Sparks, MD). DAP was provided by Merck (formerly Cubist Pharmaceuticals; Lexington, MA).

Overnight cultures grown in MH were used for inoculation to an initial optical density at 600 nm (OD_600_) of 0.05. Cultures were grown aerobically at 37°C in flasks with a 10:1 flask-to-volume ratio and with shaking at 250 rpm and supplemented when required with different concentrations of DAP and 50 μg/ml CaCl_2_. Cultures of mutants and transcomplemented VraSR strains were grown in tetracycline (5 μg/ml) and chloramphenicol (10 μg/ml), respectively. Bacterial growth was assessed by measuring OD_600_, and viability was measured by CFU/ml serial dilutions on MHA plates. Antibiotic MICs were determined using the Etest (bioMérieux, Marcy l’Etoile, France) and the broth microdilution method according to CLSI guidelines (50). ATCC 29213 was used as an internal control for MIC assays. Because CLSI has not yet established a resistance breakpoint for DAP, strains with MICs of ≥1 µg/ml were considered nonsusceptible (48). The term resistant is being used in the present study to simplify reading and understanding. Plasmid DNA was isolated from Escherichia coli strains using a QIAprep Spin miniprep kit (Qiagen, Valencia, CA) according to the manufacturer’s protocol. Plasmids were transformed into S. aureus RN4220 by electroporation using a previously described procedure (51). Plasmids were introduced in the final S. aureus strain using 80α-phage transduction (52). *In vitro* DAP-resistant mutant CB1631-R was obtained using progressive daily passages of DAP^s^ CB1631 in subinhibitory concentrations of DAP (gradient concentrations were 0, 0.06, 0.125, 0.25, 0.5, 1, 2, 4, 8, 16, and 32 µg/ml) with 50 µg/ml CaCl_2_ in 24-well plates at 37°C for 15 days. Measurement of DAP MICs was performed according to CLSI guidelines to confirm the identity of the *in vitro* mutants and their corresponding parental strains.

### Construction of DAP CB1634*+agr* complementation.

To generate the CB1634 ectopic *agr-*overexpressed construct CB1634+*agr*, the full length of *agr* was cloned into psk265 as previously described (53). Transaction of products required for complete *agr* activity, including an upstream region putative ribosomal binding site and the *agr* promoters (P2 and P3), was performed as previously described (53) (Table 1).

### Construction of *vraSR*-null mutants and complementation.

A mutant (CB1634 Δ*vraSR*::*cat*) strain was obtained by transducing the deletion *vraSR* mutant (Δ*vraSR*::*cat*) by ϕ11 phage from strain KVR (54) into DAP^r^ CB1634 (52), resulting in CB1634 *vraSR*. This mutant was transcomplemented using transduction and the pAW8 shuttle plasmid, which contained a 3.3-kb fragment corresponding to the entire *vraSR* operon (52); the CB1634Δ*vraSR*+*vraSR* strain was produced (Table 1). A similar procedure was performed into the CB5014 strain to produce a *vraSR* mutant (CB5014Δ*vraSR*) and a transcomplemented *vraSR* mutant, CB5014Δ*vraSR*+*vraSR*.

### Galleria mellonella survival assay.

Galleria mellonella infections were performed as described previously (55). Briefly, groups of 10 Galleria mellonella larvae at their last instar stage (Knutson’s Live Bait, Brooklyn, MI) were inoculated with 10 µl of bacterial suspension (∼10^7^ CFU/ml) during the last left proleg. Worms were incubated at 37°C and monitored every 24 h over a period of 5 days; those worms that did not move when touched and that were dark brown were considered dead. All trials included an uninfected control (injected with PBS). All experiments were performed in three independent replicates, and results were expressed as survival percentage versus time.

### Epithelial adhesion and invasion assays.

A549 human lung epithelial cells (ATCC CCL 185) were used for cell culture assays. First, 24-well plates were seeded with 2 × 10^5^ cells/well and incubated overnight at 37°C and 5% CO_2_. Once cells reached confluence (0.2 × 10^6^), culture medium was removed, and cells were washed with DMEM and 10% fetal bovine serum. For adhesion experiments, overnight bacterial cultures in DMEM were added to the cellular monolayer at a multiplicity of infection (MOI) of 10. After a 1-h incubation at 37°C in 5% CO_2_, nonadherent cells were removed by washing three times with PBS. For invasion experiments, overnight bacterial cultures in DMEM were added to the cellular monolayer at an MOI of 40 and incubated for 2 h at 37°C in 5% CO_2_. Medium was aspirated and replaced with DMEM and 10% fetal bovine serum containing 100 µg/ml gentamicin and 5 µg/ml lysostaphin to remove noninternalized bacteria. Cells were incubated for 2 h at 37°C and 5% CO_2_. Eukaryotic cells were detached from the wells with 0.25% Trypsin in 1 mM EDTA and lysed with 0.1% Triton X-100. Extracts were vigorously vortexed, serially diluted in PBS, and plated onto TSA. Independent experiments were performed in triplicate.

### Expression analysis using RNA-seq and qRT-PCR.

Overnight bacterial cultures grown in MH broth at 37°C and shaken at 150 rpm were diluted 1:100 in MH and incubated until they reached an OD_600_ of ∼0.5. RNAlater reagent (Sigma) was added to bacterial cell cultures to protect the cellular RNA. Total RNA extraction was performed using an RNeasy isolation kit (Qiagen), and the DNA was removed using a DNA-free DNA removal kit (Thermo Fisher Scientific). RNA concentrations were assessed by measuring absorbance at 260 and 280 nm using a NanoDrop 8000 (Thermo Fisher Scientific, Waltham, MA). For RNA-seq analysis, RNA was prepared from S. aureus CB1631 and CB1634 cells collected during the exponential phase of growth. The quality of the total RNA was determined using RNA Nano chips (Agilent Technologies, Santa Clara, CA) run with an Agilent 2100 Bioanalyzer and 2100 Expert software. The genome-wide transcript sequencing libraries were prepared according to the manufacturer’s recommendations (ScriptSeq; Epicentre) and sequenced on a MiSeq instrument (Illumina, San Diego, CA). Differential gene expression was determined using Lasergene (v14) software (DNAStar, Madison, WI) and the PATRIC web resource (56); differences of >1.5-fold and *P* < 0.05 after applying Bonferroni correction were considered significant. For qRT-PCR, real-time reverse transcription-PCR analysis was performed using a SensiFAST SYBR No-ROX one-step kit (Bioline, Taunton, MA). Probes and primers were synthetized by Eurofins Genomics (Thermo Fisher Scientific, Waltham, MA); the corresponding sequences are provided in Table 3. The level of gene expression for the studied strain compared with its parental strain (reference) was expressed as 2^−ΔΔ^*^CT^*, where *C_T_* represents the threshold cycle value, Δ*C_T_* represents the difference in threshold cycle between the target gene and the control gene (16S), and ΔΔ*C_T_* represents the difference in Δ*C_T_* between the studied strain and the parental strain. Values represent the means from three independent experiments.

**TABLE 3:** Primers and probes used in this study

Primer	Sequence (5′→3′)
agrA-F	CGCAACTGATAATATGAGGTGCTTGA
agrA-R	CAACTGGGTCATGCGAATTTCACTGC
clfB-F	GGTGGTGTAACTCTTGAATCGGAGTC
clfB-R	GGACTCAGACAGCGATTCAGATTCAG
cap5E-F	ATACGACAGAAGCGTAGAATCATTAG
cap5E-R	GTGTTGGCTTACACATATCGCCATC
hlb-F	AGCTACTCATCAACTGTTGCTG
hlb-R	GTTGCTATCATTATCGAATCCAC
Hld-F	GTTCACTGTGTCGATAATCCA
Hld-R	AGGAAGGAGTGATTTCAATGG
icaA-F	AAACTTGGTGCGGTTACAGG
icaA-R	GTAGCCAACGTCGACAACTG
psmB-F	TTATTTCAAAGGTGAGGGAGAGATTT
psmB-R	TTGTTGTGCAGCTTGCACAGT
saeR-F	AATACCATCATCAACCAGTT
saeR-R	CTCAAATTCCTTAATACGCATA
sigB-F	CTAAATCTTCGTGATGTGATTGTCG
sigB-R	AACCAATGGATTAAAGAACACCAAG
splA-F	AGGCGGAGGAAACTACGA
splA-R	ACTATCGCAAGGTCTTCT
vraSR-F	GGTGCAACGTTCCATATTGTATCATT
vraSR-R	GGCTTCAACTCATGGGCTTTGGCAA
walK-F	AAACAACTACAATCCCTTCATACTAA
walK-R	CTTGACGGTTGGCATACTCACTTAA
16S-F	TCCGGAATTATTGGGCGTAA
16S-R	CCACTTTCCTCTTCTGCACTCA
SaeR-Fw2	TTGATATCATGGTACTTGATATCA
SaeR-Rv	CTCAAATTCCTTAATACGCATA

### Hemolysis assay.

Hemolytic activity was assayed as described previously (57). Briefly, bacteria were grown overnight in TSB at 37°C with shaking at 150 rpm. The OD_600_ was measured, and the OD of bacterial cultures was adjusted to the lowest value. Supernatants were filter sterilized (0.22 µm) and incubated with equal volumes of 2% RBC solution (Hardy Diagnostics, Santa Maria, CA) for 1 h at 37°C and 5% CO_2_. Cells were centrifuged at 13.000 rpm for 10 s. Released hemoglobin was measured by determining the absorbance at 540 nm. PBS and S. epidermidis Y1 were used as spontaneous hemolysis and nonhemolytic controls, respectively. Staphylococcusaureus subsp. *aureus* ATCC 29213 was used as the beta-hemolytic control. A standard curve was performed to determine the percentage of hemolysis. Independent experiments were performed in triplicate.

### δ-Hemolysis assay.

Evaluation of δ-hemolysis production was used as an indirect test to determine *agr* functionality and was performed as described previously (58). Briefly, S. aureus RN4220 (beta-hemolytic) was streaked along the middle of a blood agar plate. Strains of interest were streaked perpendicular to RN4220 to determine presence or absence of δ-hemolysis.

### Whole-genome sequencing.

Chromosomal DNA from staphylococcal strains grown in MH overnight at 37°C was prepared using the DNeasy Blood and Tissue kit (Qiagen). Library preparation and sequencing (MiSeq; Illumina) was performed by the Epigenetics and Genomics laboratory at Weill Cornell University, New York, NY. Genomes were assembled, annotated, and analyzed for nucleotide changes using Lasergene (v14) software (DNAStar, Madison, WI) and the PATRIC variation analysis service. The S. aureus N315 sequence (GenBank accession number BA000018; PATRIC ID 158879.11) was used as the reference sequence.

### Secreted protein preparation and Western blot analysis.

Bacteria were grown in MH until reaching an OD_600_ of approximately 0.6. Then, the samples were centrifuged for 10 min at 4,000 rpm, and the supernatant was passed through 0.22-μm-pore-size membrane filters (Millex; Millipore Sigma, Burlington, MA). Samples were normalized by adjustment of the volume to equal the sample OD as previously reported (59). Samples were concentrated in Amicon 10,000-molecular-weight-cutoff centrifugal filters (Millipore Sigma) to a final volume of 40 μl. Ponceau staining was used as load control.

For Western blot analysis, 20 μg of proteins of each sample was loaded and separated using 4 to 12% SDS-PAGE electrophoresis gradient gels (ThermoFisher, Carlsbad, CA), after which they were blot transferred onto pure nitrocellulose blotting membranes (Fisher Scientific, Hampton, NH). The membranes were blocked using 5% low-fat milk in PBS. Alpha-toxin was probed with a polyclonal anti-alpha antibody (Millipore Sigma) at a 1/2,000 dilution, followed by incubation with a secondary goat anti-rabbit IgG(H+L) antibody at a 1/5,000 dilution. Protein bands were developed in autoradiography films (Denville Scientific Inc., South Plainfield, NJ).

### Murine sepsis model.

A septicemia mouse model was used to determine the role of DAP susceptibility in S. aureus pathogenicity. Groups of six SCID Beige mice (Envigo, Houston, TX) were used. Each group (*n* = 6) was inoculated via tail injection with ∼1 × 10^7^ to 2 × 10^7^ CFU of either the DAP^s^ CB5013 or DAP^r^ CB5014 MRSA strain and mutant CB5014Δ*vraSR* and transcomplemented CB5014Δ*vraSR*+*vraSR* MRSA strains grown in TSB at 37°C, 150 rpm. Mice were euthanized at 72 h postinfection. Kidneys and spleen were collected aseptically, homogenized with a homogenizer (150 Homogenizer; Fisher Scientific), serially diluted in PBS, and plated onto TSA plates to determine the number of viable staphylococci. Results were expressed as the logarithm of CFU per gram of organ (log CFU/g).

### Animal ethics statement.

All animal studies were approved by the Institutional Animal Care and Use Committee of the Houston Methodist Research Institute. To ensure protection and proper manipulation of animals, experiments were performed by trained personnel at the animal facility of the Houston Methodist Research Institute.

### Statistical analysis.

The statistical analysis was performed using GraphPad Prism 7 software. The log rank test was used to assess significant differences (*P* < 0.05) for Kaplan-Meier survival curves. The unpaired Student *t* test was used to determine significant differences (*P* < 0.05) for adhered and internalized bacteria in cell culture assays, log CFU/g in the *in vivo* mouse sepsis model, and the mRNA expression of the CB1631 and CB1634 strains from the qRT-PCR analysis. One-way ANOVA (*P* < 0.05) was used to evaluate the significance of differences in hemolysis percentage and the mRNA expression of the CB5013 and CB5014 strains from the qRT-PCR analysis. To test the normality and homoscedasticity assumptions of the ANOVA, a Shapiro-Wilk test (*P* < 0.05) and a Brown-Forsythe test (*P* < 0.05), respectively, were performed. As an *a posteriori* analysis, the Bonferroni multiple-comparison test (*P* < 0.05) was performed.
